# Identification of inhibitors of *Plasmodium falciparum *phosphoethanolamine methyltransferase using an enzyme-coupled transmethylation assay

**DOI:** 10.1186/1471-2091-11-4

**Published:** 2010-01-19

**Authors:** April M Bobenchik, Jae-Yeon Choi, Arunima Mishra, Iulian N Rujan, Bing Hao, Dennis R Voelker, Jeffrey C Hoch, Choukri Ben Mamoun

**Affiliations:** 1 Department of Internal Medicine, Section of Infectious Diseases, Yale School of Medicine, 333 Cedar St., New Haven, 06052, USA; 2 Department of Genetics and Developmental Biology, University of Connecticut Health Center, 263 Farmington Ave., Farmington, 06030, USA; 3 The Program in Cell Biology, Department of Medicine, National Jewish Medical and Research Center, 1400 Jackson St, Denver, 80206, USA; 4 Department of Molecular, Microbial, and Structural Biology University of Connecticut Health Center, 263 Farmington Ave., Farmington, 06030, USA

## Abstract

**Background:**

The phosphoethanolamine methyltransferase, PfPMT, of the human malaria parasite *Plasmodium falciparum*, a member of a newly identified family of phosphoethanolamine methyltransferases (PMT) found solely in some protozoa, nematodes, frogs, and plants, is involved in the synthesis of the major membrane phospholipid, phosphatidylcholine. PMT enzymes catalyze a three-step S-adenosylmethionine-dependent methylation of the nitrogen atom of phosphoethanolamine to form phosphocholine. In *P. falciparum*, this activity is a limiting step in the pathway of synthesis of phosphatidylcholine from serine and plays an important role in the development, replication and survival of the parasite within human red blood cells.

**Results:**

We have employed an enzyme-coupled methylation assay to screen for potential inhibitors of PfPMT. In addition to hexadecyltrimethylammonium, previously known to inhibit PfPMT, two compounds dodecyltrimethylammonium and amodiaquine were also found to inhibit PfPMT activity *in vitro*. Interestingly, PfPMT activity was not inhibited by the amodiaquine analog, chloroquine, or other aminoquinolines, amino alcohols, or histamine methyltransferase inhibitors. Using yeast as a surrogate system we found that unlike wild-type cells, yeast mutants that rely on PfPMT for survival were sensitive to amodiaquine, and their phosphatidylcholine biosynthesis was inhibited by this compound. Furthermore NMR titration studies to characterize the interaction between amoidaquine and PfPMT demonstrated a specific and concentration dependent binding of the compound to the enzyme.

**Conclusion:**

The identification of amodiaquine as an inhibitor of PfPMT *in vitro *and in yeast, and the biophysical evidence for the specific interaction of the compound with the enzyme will set the stage for the development of analogs of this drug that specifically inhibit this enzyme and possibly other PMTs.

## Background

Malaria is one of the most important parasitic diseases worldwide responsible for over 200 million clinical cases with an estimated 1 million deaths annually [1]. Malaria is caused by intraerythrocytic protozoan parasites of the genus *Plasmodium*. Of the five species infective to humans, *Plasmodium falciparum *is responsible for the highest level of mortality and morbidity [1]. The lack of an effective vaccine and the rapid rise of resistance to the most potent and affordable antimalarial drugs creates an urgent need for new therapies to prevent malaria pathogenesis and transmission. Novel strategies are now necessary to limit the appearance and spread of drug resistant malaria strains. One such a strategy involves targeting metabolic pathways proven to play an essential function in the parasite's infection and transmission. Recent studies indicate that the metabolic pathways for the synthesis of the major *P. falciparum *phospholipids are excellent targets for the development of lipid-based antimalarial therapies [2-4].

Studies in *P. falciparum *demonstrated that the synthesis of phosphatidylcholine during the intraerythrocytic life cycle of the parasite occurs via two pathways, the serine-decarboxylase phosphoethanolemine methyltransferase (SDPM) pathway and the CDP-choline pathway [2-4]. The SDPM pathway uses serine either transported from human serum or resulting from degradation of host hemoglobin as a starting precursor. Serine is first decarboxylated by a parasite serine decarboxylase to form ethanolamine. Ethanolamine is next phosphorylated by a parasite ethanolamine kinase to form phosphoethanolamine (P-EA). A parasite S-adenosylmethionine (SAM)-dependent methyltransferase, PfPMT, catalyzes a three-step methylation of P-EA to form phosphocholine [3,5,6], which is converted into phosphatidylcholine (PtdCho) via the activity of two parasite enzymes PfCCT and PfCEPT. The CDP-choline pathway uses choline transported from the host as a precursor. Choline is phosphorylated by a parasite choline kinase PfCK to phosphocholine, which is subsequently modified by PfCCT to CDP-choline and by PfCEPT to PtdCho.

The 266 amino acid PfPMT is a member of a new class of SAM-dependent methyltransferases that acts on P-EA [3]. Homologs of this enzyme are found in plants, nematodes, frogs, fish and other protozoa but not in mammals [7-11]. While phosphoethanolamine methyltransferases (PEAMT) share significant homology in their primary structure, the organization of their catalytic domains differ. The plant PEAMTs have two tandem catalytic domains, with the N-terminal domain catalyzing the methylation of P-EA to monomethylphosphoethanolamine and the C-terminal domain acting in the last two methylation reactions to form phosphocholine [7,9,10]. *C. elegans *expresses two PEAMT enzymes each containing only one methyltransferase domain located at either the N-terminus of the protein, in the case of Pmt1, or at the C-terminus of the protein in Pmt2 [8,11]. Pmt1 catalyzes only the first methylation reaction, whereas Pmt2 catalyzes the last two methylation reactions. The *Plasmodium *PfPMT is only half the size of the plant and nematode proteins, possesses a single catalytic domain, and catalyzes all three methylation steps [3]. No crystal or solution structures of these enzymes are available.

Confocal and immunoelectron microscopy studies have shown that PfPMT is expressed in the Golgi apparatus of the parasite [12]. Biochemical and genetic analyses using yeast as a surrogate system allowed identification of residues in this enzyme that play a critical role in PfPMT catalysis and substrate binding [6]. The finding that PfPMT has no homologs in mammalian databases suggested that this protein could be an ideal target for the development of novel antimalarial inhibitors targeting lipid metabolism. Interestingly, biochemical studies revealed that PfPMT activity was inhibited by S-adenosyl homocysteine (SAH) as well as by phosphocholine and its analog and anticancer drug, hexadecylphosphocholine (Miltefosine) [3]. When tested against *P. falciparum*, hexadecylphosphocholine was found to inhibit the growth of the parasite within human erythrocytes with 50% inhibitory concentrations in the low micromolar range [3]. Genetic studies in *P. falciparum *demonstrated that PfPMT plays an important role during the intraerythrocytic life cycle of the parasite [13]. Parasites lacking PfPMT display severe alterations in development, replication and survival within human red blood cells [13]. These defects were only partially complemented by choline supplementation.

Here we provide biochemical and biophysical data indicating that PfPMT activity is inhibited by amodiaquine (AQ). Using yeast as a surrogate system we show that AQ inhibits PfPMT activity and blocks PtdCho biosynthesis.

## Methods

### Materials

S-adenosylmethionine (SAM), S-adenosylhomocysteine (SAH), Phosphoethanolamine (P-Etn), Cholorquine (CQ), Amodiaquine (AQ), Hexadecyltrimethylammonium bromide (HDTA), Dodecyltrimethylammonium bromide (DDTA), 2,3-Dichloro- a- methylbenzylamine (DCMB), Chlorpromazine, and Diphenhydramine were purchased from Sigma. Miltefosine (HePC) was purchased from Cayman chemicals. SKF91488 and Tacrine were purchased from Tocris Bioscience. Recombinant SAH nucleosidase (SAHN) was purified from an E. coli strain generously given by K. Cornell (VAMC, Portland, OR, USA), as previously described [14].

### Expression and purification of recombinant PfPMT and BsAda

*P. falciparum PfPMT *cDNA cloned in the expression vector pET-15b and expressed in *E. coli *BL21-CodonPlus strain was purified as described earlier [3,6]. Expression strains were grown at 37°C in LB medium containing 100 μg/ml ampicillin and 35 μg/ml chloramphenicol. A 1 L culture of *E. coli *was grown until an A_600 _~ 0.6, and PfPMT expression was induced by addition of 1 mM isopropyl-β-D-thiogalactopyranosidase (IPTG). The cells were harvested 3 h after induction by centrifugation, and resuspended in 25 ml lysis buffer (50 mM NaH_2_PO_4_, 300 mM NaCl, 10 mM imidazole, pH 8.0) containing a protease inhibitor cocktail (complete Mini, EDTA-free, Roche Diagnostics). The cells were lysed by sonication using 10 s discontinuous cycles for three minutes on ice, and the cell debris and unbroken cells were removed by centrifugation at 10,000 rpm at 4°C for 30 min. The supernatant was directly applied to a 5 ml Ni-NTA (Qiagen) column pre-equilibrated in lysis buffer. The column was subsequently washed with 40 ml of buffer (50 mM NaH_2_PO_4_, 300 mM NaCl, 20 mM imidazole, pH 8.0) and the protein was eluted from the column in a single 15 ml fraction of buffer (50 mM NaH_2_PO_4_, 300 mM NaCl, 250 mM imidazole, pH 8.0). The protein was incubated at 4°C for 1 h in the presence of 2 mM Na_2_EDTA, followed by dialysis and concentration against HEPES (N-(2-hydroxyethyl)-piperazine-N'-2-ethanesulfonic acid) assay buffer (100 mM HEPES-KOH, pH 7.5) using Amicon Ultra centrifugal filter devices. The purity of the recombinant enzyme was examined by SDS-PAGE, and the protein concentration was measured by the method of Bradford using bovine serum albumin as a standard.

The gene encoding *Bacillus subtilis *adenine deaminase (BsAda) was amplified from genomic DNA by PCR and ligated into a pET15b plasmid vector between XhoI and BamHI sites to yield an N-terminal 6His-epitope-tagged encoding construct. The resulting plasmid was then transformed into *E. coli *BL21-CodonPlus strain for enzyme expression. The purification protocol for BsAda is similar to PfPMT purification except that the 1 hr incubation step with 2 mM Na_2_EDTA was omitted.

### PfPMT enzyme-coupled spectrophotometric assay

Assays were performed in 96-well UV-transparent plates (acrylic, non-sterile, Costar) at 37°C. Manganese sulfate (MnSO4) was added to a final concentration of 1 mM [15]. The assay mixture in 1X HEPES assay buffer (100 mM HEPES-KOH, pH 7.5) contained 200 μM SAM, 200 μM P-EA, 1 mM MnSO4, 0.5 μM BsAda, 4.72 μM SAHN and 2.5 μM PfPMT in a total volume of 200 μl. A mixture without PfPMT was pre-incubated at 37°C for 10 min and the reaction was initiated with the addition of PfPMT. Absorbance at 265 nm was continuously recorded with a UV, visible plate-reader (Synergy HT Multi-Mode Microplate Reader, Biotek) under kinetic mode. A negative control containing all the reaction components except for PfPMT was conducted on the same plate as the experimental reactions. The absorbance value of the control reaction was subtracted from the experimental absorbance values to eliminate the background signal.

### Inhibition studies

All inhibitor containing stock solutions were prepared in H_2_O, except for HDTA, which was prepared in DMSO. The final concentration of DMSO in the assays did not exceed 10%. Control experiments containing reaction components and inhibitors were performed in the absence of PfPMT to quantify the intrinsic absorbance of inhibitors at 265 nm. These inhibitors were added before the pre-incubation step. The absorbance at 265 nm for a PfPMT assay without inhibitor was assigned as 100% PfPMT activity. The absorbance values obtained for the various inhibitor concentrations were then converted into a PfPMT activity percentage and plotted against inhibitor concentration. Control experiments were performed to ensure that the inhibitors did not affect the coupling enzymes. To measure coupling enzyme sensitivity to inhibitors, 100 μM SAH was used in place of SAM and P-EA in a 200 μl reaction in the presence of the inhibitor at the highest concentration used in PfPMT assay. For Ki determination **e**nzymatic assays were performed with increasing concentrations of P-EA (50, 75, 100, 125, 150, 175, and 200 μM), 200 μM SAM, 1 mM MnSO_4_, 0.5 μM BsAda, 4.72 μM SAHN and 2.5 μM PfPMT in a HEPES assay buffer, along with the inhibitor AQ (0, 2, 4, 10 μM). A reciprocal plot was generated from the absorbance readings at 265 nm versus the P-EA concentrations for each concentration of AQ. The fitted line was extrapolated through the X axis to determine the estimated Ki.

### Yeast growth assays

BY4741-pYes2.1 (wild-type) and mutant *pem1Δpem2Δ-*pYes2.1-PfPMT yeast strains were pre-grown overnight in uracil dropout synthetic galactose (4%) (SG-ura) medium supplemented with 10 μM ethanolamine. The next day, cells were harvested by centrifugation, washed twice by resuspension in water and diluted to an A_600 _= 0.005 in fresh SG-ura medium supplemented with 100 μM ethanolamine, in the absence or presence of 10, 50, or 100 μM AQ. In a second set of experiments BY4741-pYes2.1, *pem1Δpem2Δ*-pYes2.1 and *pem1Δpem2Δ*-pYes2.1-PfPMT yeast strains were pre-grown overnight in SG-ura medium supplemented with 2 mM ethanolamine. The next day, cells were harvested by centrifugation, washed by resuspension in water and diluted to an A_600 _= 0.005 in fresh SG-ura medium lacking or supplemented with 1 mM choline in the presence of 0 or 200 μM AQ. In both sets of experiments, cells were grown at 30°C and monitored by measuring the A_600_.

### Yeast phospholipid analysis

*pem1Δpem2Δ*-pYes2.1 and *pem1Δpem2Δ-*pYes2.1-PfPMT yeast strains were pre-grown in SG-ura medium supplemented with 2 mM ethanolamine and 2 mM choline for 24 hours. The cells were harvested by centrifugation, washed twice by resuspension in water and diluted to an A_600 _= 0.03 in the SG-ura medium supplemented with 2 mM ethanolamine and grown overnight to an A_600 _~ 1.5. The cells were next harvested and the lipids extracted for two dimensional thin layer chromatography using previously described methods [5,6,16]. Lipids were stained with iodine vapor and excised from the plate for quantification by measuring phosphorus [17]. The results are shown as the percentage of total phosphorus in each phospholipid fraction.

### NMR experiments

For NMR analysis, *E. coli *cells expressing 6his-epitope-tagged PfPMT were grown in minimal medium (M9) containing ^15 ^N-ammonium chloride. The purification of the recombinant enzyme followed the same procedure described above except for the addition of a second step of purification through a Superdex G75 gel filtration column in a buffer containing 50 mM HEPES, 50 mM NaCl, 5 mM DTT pH 6.9. The sample was concentrated and D_2_O was added to a final concentration of 7% (v/v). Amodiaquine stock solution was prepared in ddH_2_O due to its low solubility. Serial dilutions were prepared and 5 μl of the diluted samples were added to the NMR sample containing 310 μM of purified PfPMT. The AQ concentration was determined spectrophotometrically using extinction coefficients ε_238 _= 27 × 10^3^M^-1^cm^-1^, ε_251 _= 23 × 10^3^M^-1^cm^-1 ^and ε_341 _= 19 × 10^3^M^-1^cm^-1 ^[18]. The PfPMT concentration was determined using the Edelhoch method [19-21]. Standard ^1^H-^15^N HSQC experiments were performed on ^15^N-labeled PfPMT using a Varian Inova spectrometer operating at a ^1^H frequency of 600 MHz. Data were processed using nmrPipe [22] and analyzed using Sparky [23] or the Rowland NMR Toolkit http://rnmrtk.uchc.edu.

## Results

### Coupling PfPMT activity to that of SAH nucleosidase and adenine deaminase

**Figure 1:**
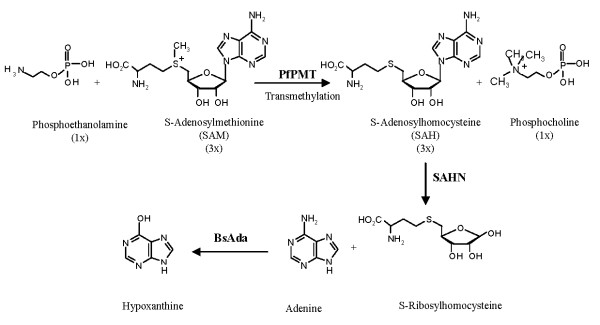
**Schematic description of the enzyme-coupled assay for measuring PfPMT activity**. PfPMT catalyzes the conversion of 1 molecule of P-EA and 3 molecules of SAM to form 1 molecule of phosphocholine and 3 molecules of SAH. SAH is hydrolyzed to adenine and S-ribosylhomocysteine via SAHN nucleosidase. The deamination of adenine into hypoxanthine by adenine deaminase is associated with a decrease in absorbance at 265 nm that can be monitored continuously using UV plate reader.

**Figure 2:**
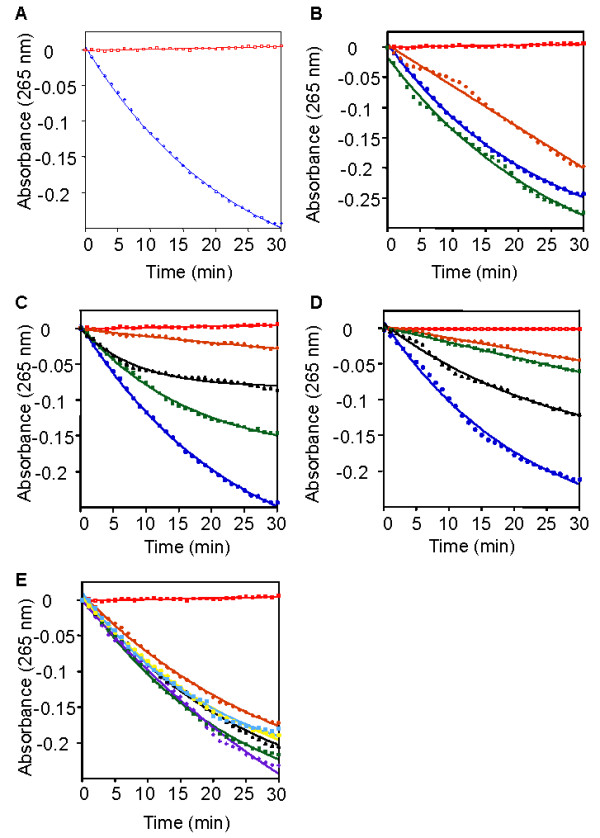
**Spectrophotometric analysis of phosphoethanolamine methyltransferase activity**. (A) PfPMT-catalyzed methylation of P-EA. Reaction mixtures contained 200 μM SAM, 200 μM P-EA, 1000 μM MnSO_4_, 0.5 μM BsAda, 4.72 μM SAHN and 0 μM (red) or 2.5 μM (blue) of purified PfPMT enzyme in 100 mM HEPES assay buffer pH 7.5. The decrease in absorbance was monitored at 265 nm. The reactions components were kept the same as stated above in panels B-E, except when noted. (B) Dependence of the rate of PfPMT-catalyzed reaction on different concentrations of the coupling enzymes. Reactions contained BsAda 0.25 μM and SAHN 2.13 μM (orange), 0.5 μM BsAda, 4.72 μM SAHN (blue) and without PfPMT (red), and BsAda 1 μM and SAHN 8.5 μM (green). (C) Dependence of the rate of PfPMT-catalyzed reaction on different concentrations of SAM and P-EA. Reactions contained P-EA 50 μM and SAM 150 μM (orange), P-EA 100 μM and SAM 100 μM (black), p-Etn 150 μM and SAM 150 μM (green), P-EA 200 μM and SAM 200 μM (blue), and without PfPMT (red). (D) Effect of PfPMT concentration on its activity. Reactions contained 0 μM PfPMT (red), 312.5 nM PfPMT (orange), 625 nM PfPMT (green), 1.25 μM PfPMT (black), and 2.5 μM (blue). (E) Effect of the pH and buffer composition on PfPMT activity. Reaction mixtures contained 200 μM SAM, 200 μM P-EA, 1000 μM MnSO_4_, 0.5 μM BsAda, 4.72 μM SAHN and 0 μM (red) or 2.5 μM (black), of purified PfPMT enzyme in 100 mM HEPES assay buffer pH 7.5. Reactions also contained 100 mM HEPES assay buffer pH 7 (green), and pH 8 (cyan), as well as 100 mM Tris-HCl assay buffer pH 7 (orange), pH 7.5 (purple), and pH 8 (yellow). Results are representative of three independent experiments.

To identify new inhibitors of PfPMT we adapted an assay that couples the SAM-dependent transmethylation of P-EA by PfPMT to the activity of two enzymes SAH nucleosidase (SAHN) and adenine deaminase (BsAda) (Fig. 1). PfPMT activity results in the production of SAH and phosphocholine. The second enzyme SAHN catalyzes the conversion of SAH into adenine and S-ribosylhomocysteine. Adenine is converted into hypoxanthine by the third enzyme, BsAda, thus producing a decrease in absorbance at 265 nm (Fig. 2A). As shown in Fig. 2A, addition of PfPMT resulted in a time-dependent decrease in absorbance at 265 nm over a 30 min period, whereas no change in absorbance occurred when the enzyme was omitted. To demonstrate that the measured rate of the coupled reactions was determined solely by PfPMT, the enzyme activity was investigated in the presence of a fixed PfPMT concentration but with varying concentrations of SAHN or BsAda. As shown in Fig. 2B, changing the concentrations of the coupling enzymes had little or no effect on the overall rate of the reactions. The effect of increasing concentrations of the substrate P-EA and co-substrate SAM on PfPMT activity was also measured. PfPMT activity increased proportionally to the concentration of these substrates, with optimal activity measured when the substrate and co-substrate concentrations were at 200 μM (Fig. 2C). Higher concentrations of P-EA only slightly affected the activity of the enzyme, whereas high SAM concentrations inhibited the enzyme (data not shown). Under optimal conditions, we also examined the effect of PfPMT concentration on the rate of the reaction. As shown in Fig. 2D, increasing the concentration of PfPMT resulted in a proportional increase in activity reflected by a decrease in absorbance over time. Above this range, the enzyme activity started to deviate from linearity (data not shown). We also tested the effect of different buffers at pH 7, 7.5 and 8 on PfPMT activity. These factors were found to have little effect on PfPMT activity over time (Fig. 2E).

#### Application of the PfPMT spectrophotometric assay for screening inhibitors of the enzyme

**Figure 3:**
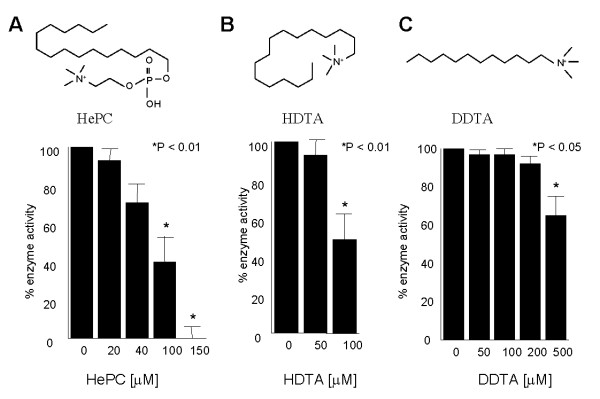
**Inhibition of PfPMT by quaternary amines**. Effect of increasing concentrations of hexadecylphosphocholine (HePC) (A), hexadecyltrimethylammonium bromide (HDTA) (B) and dodecyltrimethylammonium bromide (DDTA) on PfPMT activity. The assay was performed as described in Experimental Procedures. The data are the means +/- S.D. for three independent experiments. Statistically significant data with a P < 0.05 is indicated with an asterisk.

To assess the applicability of the enzyme-coupled assay for screening inhibitors of PfPMT, the effects of some known or potential inhibitors of this enzyme were examined. As most drug screens are performed in the presence of DMSO, we first tested the effect of this agent on PfPMT activity. DMSO up to 10% had no effect on PfPMT activity (data not shown). The hexadecylphosphocholine analog, miltefosine (HePC), was previously reported to inhibit PfPMT activity *in vitro *and *P. falciparum *proliferation inside red blood cells with similar efficacy [3]. Using the enzyme-coupled spectrophotometric assay, HePC was found to inhibit PfPMT activity with 100 μM of HePC reducing PfPMT activity by ~60% and 150 μM of the compound resulting in a complete loss of activity (Fig. 3A). As a control, an assay in which PfPMT, SAM and P-EA were omitted and replaced by their product SAH was performed in the absence or presence of 200 μM of HePC. Under these conditions HePC had no effect on the production of hypoxanthine over time, suggesting that neither SAHN nor BsAda was inhibited by this compound. Two compounds that are structurally related to HePC, hexadecyltrimethylammonium (HDTA) and dodecyltrimethylammonium (DDTA) that are known to have potent antimalarial activity *in vitro *and *in vivo *[24-26] were also tested to determine their inhibitory activity against PfPMT. HDTA was found to have an inhibitory activity similar to that of HePC with ~50% inhibition of PfPMT activity observed at 100 μM of the compound (Fig. 3B). DDTA, however, had no effect against the enzyme when used at concentrations of up to 100 μM and only modest inhibitory activity at 500 μM, inhibiting 25% of PfPMT activity at this concentration (Fig. 3C).

### Inhibition of PfPMT activity by amodiaquine *in vitro *and in yeast

**Figure 4:**
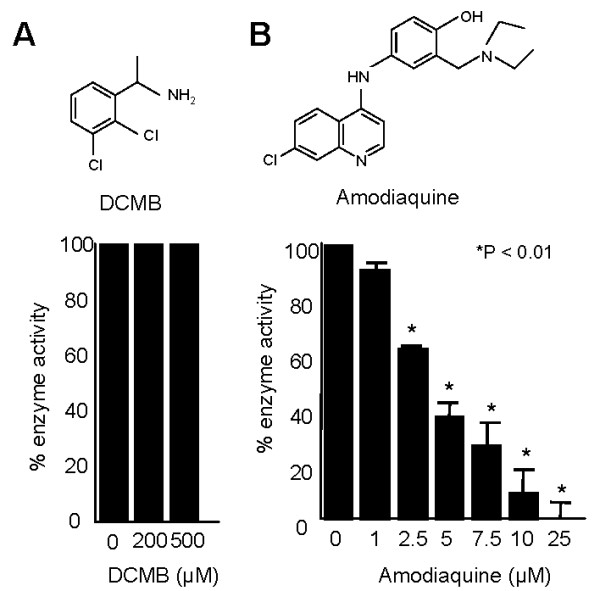
**Amodiaquine inhibits purified PfPMT activity**. Effect of increasing concentrations of DCMB (A) and amodiaquine (AQ) (B) on PfPMT activity. The assay was performed as described in Methods. The data are the means +/- S.D. for three independent experiments. Statistically significant data with a P < 0.01 is indicated with an asterisk.

**Figure 5:**
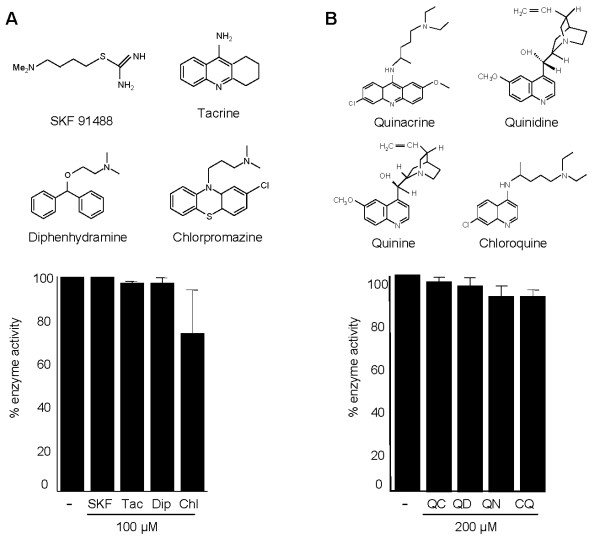
**Effect of HNMT inhibitors and antimalarial aminoquinolines and amino alcohols on PfPMT activity**. (A) Effect of the HNMT inhibitors SKF91488 (SKF), tacrine (Tac), diphenhydramine (Dip) and chlorpromazine (Chl) on PfPMT activity. (B) Effect of chloroquine (CQ), quinacrine (QC), quinidine (QD) and quinine (QN) on PfPMT activity. The data are the means +/- S.D. for three independent experiments.

**Figure 6:**
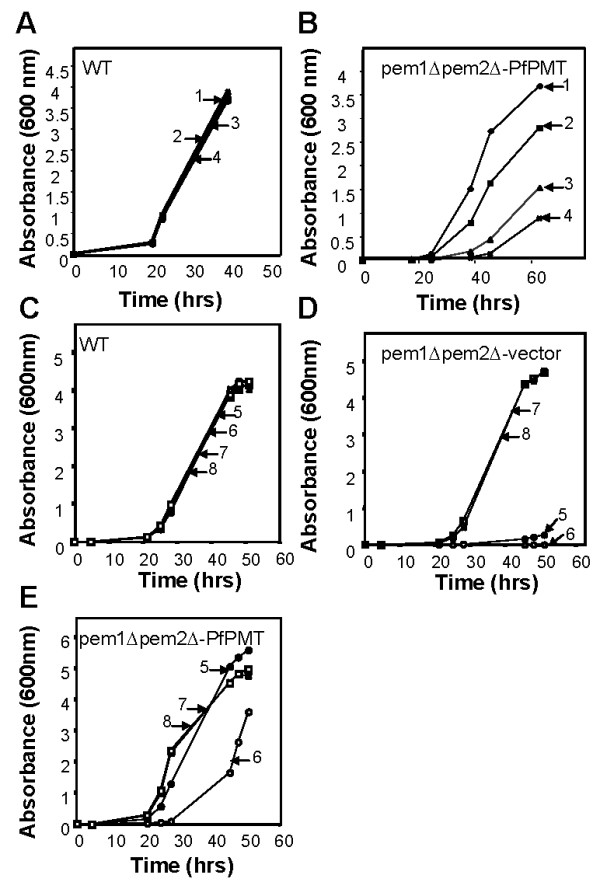
**Amodiaquine inhibits PfPMT function in yeast**. Growth curves of wild-type (BY4741-pYes2.1) (A) and *pem1Δpem2Δ-*PfPMT (B) strains grown in minimal medium containing 4% galactose and 100 μM ethanolamine in the presence of 0 μM (1), 10 μM (2), 50 μM (3), or 100 μM (4) AQ. (C-E) Growth curves of wild-type (BY4741-pYes2.1) (C), *pem1Δpem2Δ*-pYes2.1 (D) and *pem1Δpem2Δ*-PfPMT (E) yeast strains grown in minimal medium containing 4% galactose and 2 mM ethanolamine in the presence of 0 μM AQ (5), 200 μM AQ (6), 200 μM AQ and 1 mM choline (7), or 1 mM choline (8).

In order to identify possible lead inhibitors of PfPMT, we examined the effect of drugs known to inhibit other SAM-dependent methyltransferases. The 2,3-dichloro-methylbenzyl-amine (DCMB), a known inhibitor of the human phenylethanolamine-N-methyltransferase enzyme [27], which catalyses the terminal step in epinephrine biosynthesis had no effect on PfPMT activity even at 500 μM (Fig. 4A). Conversely, AQ, a known inhibitor of histamine methyltransferases (HNMT) and a potent antimalarial drug [28], was found to be a good inhibitor of the enzyme with 60% inhibition at 5 μM (Fig. 4B). None of these concentrations of AQ inhibited the coupling enzymes used in the PfPMT assay (data not shown). The finding that AQ inhibits PfPMT led us to investigate the effect of four other HNMT inhibitors, SKF91488, Tacrine, Chlorpromazine and Diphenhydramine. None of these compounds had a significant effect on PfPMT activity (Fig. 5A). Furthermore, none of the compounds inhibited the growth of *P. falciparum *within human erythrocytes at a concentration as high as 1 μM (data not shown). The specificity of inhibition of PfPMT by AQ was further demonstrated using its analog and antimalarial drug, chloroquine (CQ). At 200 μM concentration, CQ had no effect on PfPMT activity (Fig. 5B). Other aminoquinolines and amino alcohols, quinacrine, quinidine and quinine, known for their potent antimalarial activity, had no effect on PfPMT activity at concentrations up to 200 μM (Fig. 5B), suggesting that AQ inhibition of PfPMT is specific. In order to examine AQ inhibition of PfPMT *in vivo *we used yeast as a surrogate system. Yeast is a particularly attractive system for this analysis because, unlike *P. falciparum*, it is not sensitive to AQ and lacks phosphoethanolamine methyltransferases. We have previously shown that a codon-optimized *P. falciparum PfPMT* gene complements the choline auxotrophy of the yeast *pem1Δpem2Δ *mutant, which lacks the two phospholipid methyltransferases, Pem1p and Pem2p, and thus is unable to synthesize PtdCho from PtdEtn [29,30]. In the complemented strain, PfPMT restores PtdCho by providing phosphocholine following P-EA transmethylation [5,6]. Examination of the growth of wild-type yeast cells in media lacking or containing choline, and supplemented with either 100 μM or 2 μM ethanolamine (Fig. 6A and 6C), and in the absence or presence of AQ demonstrated no effect of this compound at concentrations up to 200 μM. Unlike *pem1Δpem2Δ*, which did not grow on medium containing ethanolamine but lacking choline (Fig. 6D, curve 5 & 6), *pem1Δpem2Δ *strains complemented with PfPMT grew on media lacking choline and their growth rate was significantly influenced by the availability of ethanolamine with the highest cell density reached in the presence of 2 μM ethanolamine (Fig 6E, curve 5 & 6). Interestingly, the growth of *pem1Δpem2Δ*+PfPMT was dramatically inhibited when AQ was added to the culture medium (Fig. 6B). AQ inhibited the growth of *pem1Δpem2Δ*+PfPMT strains in a concentration dependent manner with 100 μM drug reducing growth by 76% in medium containing 100 μM ethanolamine after 60 h (Fig. 6B). These results thus demonstrate a direct inhibition of PfPMT by AQ *in vivo*. Addition of choline to the culture medium of *pem1Δpem2Δ*-PfPMT cells resulted in complete resistance of these cells to AQ (Fig. 6E, curve 7 & 8), suggesting that the inhibition of growth was dependent on the essential function of PfPMT for survival in the absence of exogenous choline. As a control, the *pem1Δpem2Δ *mutant harboring an empty vector did not grow in the absence of choline and was resistant to AQ when choline was added (Fig. 6D).

**Figure 7:**
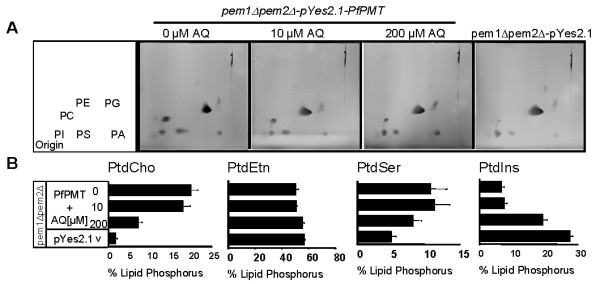
**Amodiaquine reduced PfPMT-dependent PtdCho levels in yeast**. (A) Phospholipid analysis of *pem1Δpem2Δ*-pYes2.1 and *pem1Δpem2Δ*-PfPMT strains grown in minimal medium containing 4% galactose and 2 mM ethanolamine. The lipids were extracted, separated by 2-D TLC and stained with iodine vapor. (B) Each lipid was recovered from the TLC plate and quantified by measuring phosphorous. The graph is the percentage of total lipid phosphorous in each lipid fraction. PtdCho-phosphatidylcholine; PtdEtn- phosphatidylethanolamine; PtdSer- phosphatidylserine; PtdIns- phosphatidylinositol. The data are represented as the means +/- S.D. of three independent experiments.

To demonstrate that the inhibition of the growth of *pem1Δpem2Δ*+PfPMT by AQ was due to the inhibition of the synthesis of PtdCho from ethanolamine, the synthesis of the major phospholipids PtdCho and PtdEtn of yeast membranes was examined in the absence or presence of AQ. Consistent with previous findings [5,6], *pem1Δpem2Δ *cells harboring an empty vector produced ~3% of total phospholipid as PtdCho after 5 to 6 generations of growth in the choline deficient medium, whereas those expressing PfPMT produced ~18% PtdCho (Fig. 7). Addition of AQ to *pem1Δpem2Δ *cells expressing PfPMT resulted in a concentration dependent decrease in PtdCho levels with ~15% produced at 10 μM and ~5% produced at 200 μM AQ (Figs. 7A and 7B). These findings further demonstrate the specific inhibition of PtdCho biosynthesis by this compound. The depletion of PtdCho effected by genetic manipulation or AQ treatment was partially compensated by increased levels of PtdIns (Fig. 7).

### Structural analysis of the interaction between PfPMT and amodiaquine

**Figure 8:**
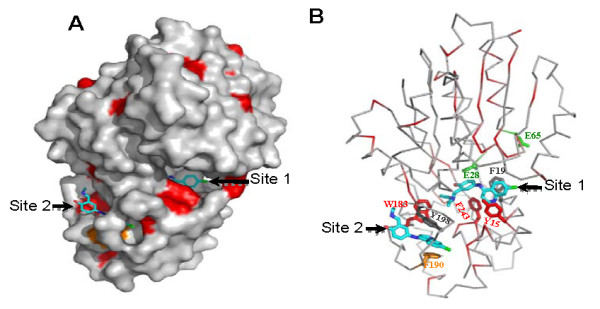
**Prediction of amodiaquine-interacting residues on PfPMT using HNMT structure**. (A) Molecular surface representation of HNMT. Red solvent-accessible surfaces identify invariant residues between HNMT and PfPMT. Phe190 is colored in orange. Two AQ molecules are shown as stick with the carbon atoms depicted in cyan, nitrogen in blue, oxygen in red, and chlorine in green. (B) The Cα trace of HNMT in a similar orientation as (A). The AQ-interacting residues are shown as stick forms. The HNMT residues, corresponding to the nine residues of PfPMT whose chemical shifts are perturbed in response to AQ binding, are shown in green. Figures were prepared by using Pymol [56].

**Figure 9:**
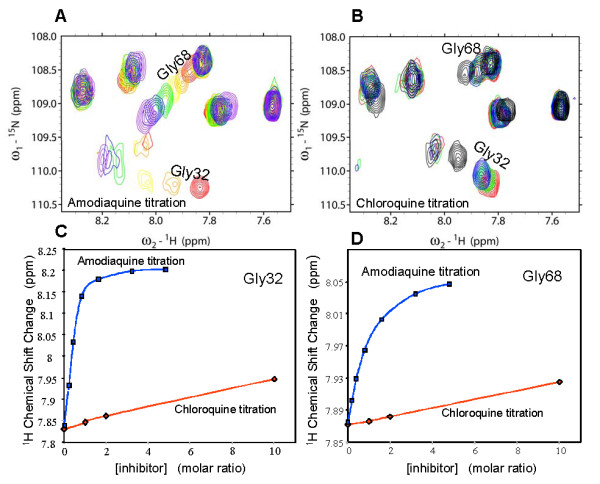
**NMR analysis of PfPMT-amodiaquine interaction**. The glycine region of the 1H-15N HSQC spectra of PfPMT (0.3 mM), titrated with AQ (A) or CQ (B). The different colors indicate the inhibitor concentrations as follows: red- no inhibitor, orange- 1:4, yellow- 1:2, green- 1:1, blue- 2:1, violet- 3:1, black- 10:1. (C and D) Inhibitor titration plots for Gly32 and Gly68 (derived from A and B) showing the difference in binding between AQ and CQ. The overlay of the full HSQC spectrum in the absence or presence of AQ is shown in Additional File 1, Fig. S1.

Co-crystallization studies of human HNMT with AQ indicated that two molecules of AQ were bound per HNMT molecule [31]. One occupies the active site pocket (Site 1; Figs. 8A and 8B) and was proposed to competitively inhibit histamine binding, and the other occupies a deep pocket representing an uncompetitive component (Site 2; Figs. 8A and 8B) [31]. To characterize the nature of the inhibition of PfPMT by AQ and to calculate the inhibition constant, PfPMT activity was determined in the presence of increasing concentrations of P-EA and increasing concentrations of the inhibitor. These studies, however, did not allow distinction between competitive and noncompetitive inhibition. To further explore the interaction of AQ with PfPMT, we performed NMR studies of the enzyme with varying concentrations of AQ. The ^1^H-^15^N HSQC spectra revealed that of the 266 residues of PfPMT only about 20 residues showed a substantial change in chemical shift as a function of increasing concentrations of AQ (Fig. 9A and Additional file 1, Fig. S1). This demonstrates direct and site-specific binding of AQ to PfPMT. We were able to tentatively assign 9 of the 20 resonances exhibiting shifts upon AQ binding. Mapped onto corresponding residues in the HNMT structure (via ClustalW sequence alignment), 8 of the 9 residues were found to be proximal to the two AQ binding sites in HNMT. The 9th residue is proximal to residues that are in contact with an AQ binding site. Nonspecific changes involving many residues that are observed at high AQ concentrations appear to reflect biophysical changes in solution properties due to a concentrated co-solute.

We also performed NMR titrations using CQ (Fig. 9B). While the effects of CQ are similarly restricted to about 20 residues, indicating site-specific binding, the affinity of CQ for PfPMT is much lower than that of AQ. Complete characterization of CQ binding by NMR is hampered by nonspecific effects that begin to dominate at concentrations above 3 mM (molar ratio of 10 in Fig. 9). Detailed comparison of the concentration dependence of the chemical shifts for two residues, tentatively assigned to Gly32 and Gly68, revealed the difference in the affinities of AQ and CQ for PfPMT. For AQ, the chemical shift changes approached an asymptotic value approximately exponentially, consistent with all binding sites occupied around a 3:1 molar ratio (Figs. 9C and 9D). In contrast, the chemical shifts of Gly32 and Gly68 changed only linearly up to a molar ratio of 10:1. The fact that the same residues are perturbed in response to both AQ and CQ suggest that they interact with the same site on PfPMT, but the different concentration dependence suggests that the affinity of CQ for PfPMT is at least an order of magnitude lower than that of AQ.

## Discussion

Methyltransferases represent a large group of enzymes divided into 160 classes (EC 2.1.1.1 to EC2.1.1.160). These enzymes catalyze the methylation of a large number of different substrates including DNA, RNAs, phospholipids, fatty acids, inositol, phosphoethanolamine, phenylethanolamine, tocopherol, catechol and histamine. Although some methyl donor molecules such as S-methylmethionine, betaine and folate can be used, the majority of biologically active methyltransferase enzymes utilize SAM as a methyl donor [32]. SAM-methyltransferases are recognized by the primary structure and topology of the core fold containing the SAM binding domain [32]. These enzymes play important functions in various biological systems and have been linked to different human diseases such as Alzheimer's disease, attention deficit disorder and pre-eclampsia [33-35]. Phosphoethanolamine methyltransferases (PMTs) represent a recently identified class of enzymes found mostly in plants, nematodes, frogs, fish and some species of protozoa. Their absence in humans and the finding that these enzymes play important physiological functions make them good targets for the development of novel inhibitors to treat worm and protozoan parasitic diseases [3,8,11]. The use of radiolabeled SAM to assay PMTs *in vitro *makes it difficult to perform large-scale screenings of available chemical libraries to search for inhibitors of these enzymes. Here we have modified a continuous coupled assay previously described by Dorgan et al [36] for the rat arginine N-methyltransferase 1 (PRMT1) to measure PfPMT activity *in vitro *and demonstrated its feasibility for drug inhibition assays using HePC, which was previously shown to inhibit PfPMT with a radioactivity-based assay [3]. In this report, PfPMT activity was inhibited ~60% at a concentration of 100 μM of HePC and 150 μM of this compound resulted in a complete loss of activity. The enzyme-coupled assay was also useful in identifying HDTA as an inhibitor of PfPMT activity. HDTA was found to have an inhibitory activity similar to that of HePC with ~50% inhibition of PfPMT activity observed at 100 μM of the compound. HDTA inhibits the growth of *P. falciparum *in culture with an IC_50 _of 2.1 μM [24]. Using this assay we have also identified AQ as an inhibitor of PfPMT. In addition to its antimalarial activity, AQ is also known as a potent inhibitor of the human histamine N-methyltransferase (HNMT) [31]. Other more potent inhibitors of HNMT such as SKF91488, tacrine, diphenhydramine and chlorpromazine [37-39] had no effect on PfPMT, suggesting that the interaction between PfPMT and AQ is unique.

Amodiaquine is a structural analog of CQ. Analysis of uncomplicated *falciparum *malaria cases in Africa suggested that AQ is more effective in clearing parasites, including CQ resistant strains, and resulted in better clinical recovery compared to CQ [40-42]. Amodiaquine is used mostly in combination therapy with artesunate and sulfadoxine-pyrimethamine to treat malaria infections, especially those caused by CQ resistant strains [43]. Following oral administration, AQ is detected in the plasma between 30 min and 8 h, but is rapidly converted to desethylamodiaquine (AQm) with a peak plasma concentration of 181 ± 26 ng ml^-1 ^[44]. In the liver AQ is converted to AQm by the polymorphic P450 isoform CYP2C8 [45]. Up to 96 hours after administration, AQm could be detected in the plasma, a property that made AQ an ideal compound in combination therapy [46]. *In vitro *both AQ and AQm inhibit parasite intraerythrocytic growth with IC_50 _values of 18.2 and 67.5 nM, respectively [47]. The modes of action of AQ and AQm are unknown. Field studies suggest that low levels of resistance to AQ associate with mutations in the *PfCRT *and *PfMDR1 *genes, whereas high AQ resistance involves unknown mechanisms [48]. These findings suggest that AQ might have CQ like properties in the food vacuole as well as novel activities that remain to be elucidated. Interestingly, whereas AQ inhibited PfPMT activity, CQ did not. PfPMT activity was also not affected by the antimalarials quinine, quinidine and quinacrine at concentrations as high as 200 μM. The specificity of inhibition by AQ was demonstrated *in vivo *by using yeast as a surrogate system. In yeast, the growth of mutants that rely on PfPMT for survival was dramatically altered in the presence of this compound. Consistent with this phenotype, the synthesis of PtdCho in these complemented cells was strongly inhibited by AQ. Surprisingly, the amplitude of inhibition of *pem1Δpem2Δ+PfPMT *cells by AQ was dependent on the concentration of ethanolamine in the culture medium. This is likely due to the fact that these complemented cells grow faster on medium containing high concentrations of ethanolamine as a result of increased substrate availability for PfPMT.

Recent genetic studies have shown that PfPMT plays an important function during *P. falciparum *intraerythrocytic development, multiplication and survival [13], and an essential role in sexual differentiation (Bobenchik et al., unpublished data). Interestingly, silencing of the *A. thaliana *PEAMT results in multiple morphological phenotypes, including pale-green leaves, early senescence, temperature-sensitive male sterility and increased sensitivity to salts [49]. Furthermore, characterization of an *A. thaliana *mutant with the T-DNA inserted at the At3g18000 locus (XIPOTL1), which encodes PEAMT revealed severe alterations in root developmental and induces cell death in root epidermal cells [50]. In *C. elegans*, studies aimed at silencing the PEAMT genes *PMT-1 *and *PMT-2 *demonstrated an essential function of these genes in worm, growth, development and survival [8,11]. Altogether, these genetic studies validate PEAMT enzymes as possible targets for the development of inhibitors that could be used for the treatment of specific human and veterinary protozoa and nematode related infections as well as in agriculture.

*P. falciparum *parasites lacking PfPMT were, however, equally sensitive to AQ as wild-type parasites in medium containing choline. This result suggests that PfPMT is not the sole target of AQ in choline-containing medium. Our finding that AQ inhibits PfPMT activity, and the fact that this compound also inhibits other eukaryotic methyltransferases, suggest that it may exert its antimalarial activity by targeting several parasite methyltransferases. Several studies have suggested that aminoquinolines may exert their antimalarial activity by interfering with biological functions other than heme detoxification in the digestive vacuole. Famin and Ginsburg reported inhibition of parasite's 6-phosphogluconate dehydrogenase by CQ [51]. Sharma and Mishra reported inhibition of parasite's tyrosine kinase by this compound [52]. Recently, CQ was reported to inhibit vesicular trafficking within the parasite [53], and to induce proteome oxidative damage [54]. More recent studies aimed at investigating the stage specificity of CQ and comparing the effect of continuous vs bolus dosing on *P. falciparum *strains revealed that rings (prior to the formation of the digestive vacuole) and schizont stage parasites (after hemoglobin degradation and heme detoxification activities have plateaued) were equally sensitive to this compound [55]. The bolus dosing concentrations of CQ used by Gligorijevic and colleagues are in the range of the compound's peak plasma concentration (low micromolar range) [55]. Together these studies suggest that heme detoxification, although important, is unlikely to be the only target of CQ. Although, the stage specificity and the effect of bolus dosing of AQ on *P. falciparum *have not been investigated, our results indicate that at its peak plasma concentration, AQ is likely to inhibit PfPMT and possibly other parasite methyltransferases. Future studies will aim to identify *Plasmodium *methyltransferases inhibited by AQ and to characterize the mechanism of sensitivity and resistance to AQ. From a chemistry standpoint, AQ represents an excellent lead compound for the rational design of better inhibitors of PfPMT and other parasite methyltransferases as well as plant and nematode PMTs.

Horton and coworkers have previously shown that AQ acts both as a competitive and a noncompetitive inhibitor of human HNMT [31]. The crystal structure of the HNMT-AQ complex [31] revealed that one AQ molecule binds to the histamine-binding site (Site 1), while the second one tucks into an adjacent pocket on the outer surface of the protein (Site 2) (Fig. 8A). Both quinoline rings fit into a sandwich-like structure formed by the aromatic side chains of Tyr15 and Phe19, and Phe190 and Tyr198, respectively (Fig. 8B). The side chains of Trp183 and Phe243 also contribute to the stabilization of the branched alkylamino tail of the AQ in the histamine-binding site [31]. It should be noted that the branch structure of the second AQ is disordered in the outer-surface pocket, suggesting a weaker binding interaction. The strong hydrophobic interactions between AQ and the aromatic side chains of the enzyme have been proposed to account for the affinity and specificity of the inhibitor for HNMT [31]. Sequence alignment analysis of HNMT and PfPMT shows that Tyr15, Trp183, and Phe243 are conserved in PfPMT, while Phe190 is substituted by tyrosine (Fig. 8B and Additional file 2, Fig. S2). This structural conservation is significant, as the two proteins have an overall 14% pairwise sequence identity. The majority of the other invariant residues are within the classic SAM-dependent methyltransferase fold in HNMT (Fig. 8B and Additional file 2, Fig. S2). Our inhibition studies revealed that whereas AQ inhibited PfPMT activity, CQ did not. PfPMT activity was also not affected by the aminoquinolines, quinine, quinidine and quinacrine at concentrations as high as 200 μM, or by the histamine methyltransferase inhibitors SKF91488, diphenhydramine and tacrine at concentrations as high as 100 μM. Unlike AQ, diphenhydramine and tacrine have been shown to interact only with Site 1 of HNMT. If this property is also valid in the case of PfPMT, it may account for the difference in inhibition of PfPMT activity between AQ and other aminoquinolines. The NMR studies reported here confirmed the site-specific binding of AQ to PfPMT, as less than 10% of residues in PfPMT showed a significant change in chemical shift in the presence of the compound. Nine of these residues have thus far been assigned. Six of those (corresponding to Glu28, Ser26, Ala63, Glu65, Ile66 and Met36 in HNMT) are proximal to the histamine-binding site (Site 1), while the 7th (Leu261 in HNMT) is proximal to Site 2. The 8th (Cys196 in HNMT) is located between the two sites. The last residue (Leu108 in HNMT) is further from Site 1, but is proximal to residues in contact with this site, and could be a relayed effect. The three residues (Gly68, Gly32 and Gly40 corresponding to Glu65, Glu28 and Met36 in HNMT) with the largest shift changes are closest to site 1. The binding of AQ to the free enzyme suggests that the inhibition is not uncompetitive, i.e. via binding to the enzyme-substrate complex, and therefore either competitive or noncompetitive. The NMR experiments at this stage do not definitively reveal whether AQ binds competitively to the substrate binding site. NMR studies to determine the structure of PfPMT alone and in combination with AQ are underway. They will address the question of noncompetitive vs. competitive inhibition, and should shed new light on the interaction between the enzyme and the inhibitor. However, based on our preliminary assignment of two of the nine assigned residues, Gly32 and Gly68, and the known structure of HNMT, we can postulate a structural hypothesis consistent with the NMR results for Gly32 and Gly68. Gly32 and Gly68 of PfPMT correspond to Glu28 and Glu65 of HNMT (Fig. 9). Glu28 lies at the bottom of the cleft comprising site 1 of HNMT. Glu65 also lines the surface of the cleft, closer to the enzyme surface. The similarity of the chemical shift perturbation curves (to within the experimental uncertainty of ~ 0.006 ppm) for Gly32 and Gly68 is thus consistent with binding at site 1. Despite the low sequence homology between PfPMT and HNMT, these data suggest that PfPMT and HNMT nevertheless exhibit structural homology, and are consistent with two AQ binding sites. The proximity of the two binding sites suggests that titration data might reflect coupling between sites. Indeed, we do not observe partitioning of the titration data into two distinct classes, as would be expected for independent binding sites.

## Conclusions

The enzyme-coupled assay adapted for PfPMT is a simple and reliable method for measuring PfPMT activity and screening for specific inhibitors of this enzyme. The identification of AQ as an inhibitor of PfPMT may help in the future design of compounds that specifically inhibit this enzyme and possibly other PMTs.

## Abbreviations

AQ: amodiaquine AQm: desethylamodiaquine CQ: chloroquine PtdCho: phosphatidylcholine; PtdEtn: phosphatidylethanolamine; PtdSer: phosphatidylserine; P-EA: phosphoethanolamine; PMT: phosphoethanolamine methyltransferase; SDPM: serine decarboxylase phosphoethanolamine methyltransferase; CDP: citidylyldiphosphate; PfCCT: CDP-choline cytidylyl-transferase; PfCEPT: CDP-diacylgylcerol-choline phosphotransferase; SAM: s-adenosylmethionine; TLC: thin layer chromatography; SAH: s-adenosylhomocystein; HNMT: histamine methyltransferase; NMR: nuclear magnetic resonance; SAHN: SAH nucleosidase.

## Competing interests

The authors declare that they have no competing interests.

## Authors' contributions

AB optimized the enzyme coupled assay, performed the enzyme inhibitor studies, prepared all the figures and assisted in the writing and editing of the manuscript and the revisions. AM contributed to the initial development of the enzyme coupled assay and cloning of the BsADA used in the study. She also helped in the design of experiments and writing of the manuscript. JC and DV conducted the yeast growth assays and the yeast phospholipid analyses. IR, BH and JH contributed to the structural modeling and NMR analysis of PfPMT-amodiaquine interaction and helped in the writing and editing of the manuscript. CBM conceived, established the experimental framework of the study, analyzed the data and contributed to the writing and editing of the manuscript. All authors read and approved the final manuscript.

## Supplementary Material

Additional file 1 **Fig. S1**. Overlay of the full ^1^H-^15^N HSQC spectra of PfPMT in the absence (red) or presence of 0.06 (orange), 0.12 (yellow), 0.25 (green), 0.5 (blue) and 1 mM (purple) of AQ. Click here for file

Additional file 2 **Fig. S2**. Sequence alignment of HNMT and PfPMT. Residues that are identical, conserved, and semi-conserved are indicated by asterisk, colon, and period, respectively. The AQ-interacting residues are colored as in Fig. 8B. Phe19 and Tyr198 of HNMT and their corresponding residues in PfPMT are shown in italics and bold. Click here for file
