# Supplementary figures and images for: Identification of inhibitors of Plasmodium falciparum phosphoethanolamine methyltransferase using an enzyme-coupled transmethylation assay
Source: BMC Biochem. 2010 Jan 19;11:4. doi: 10.1186/1471-2091-11-4 (PMC2824672; doi:10.1186/1471-2091-11-4)

## Slide 1
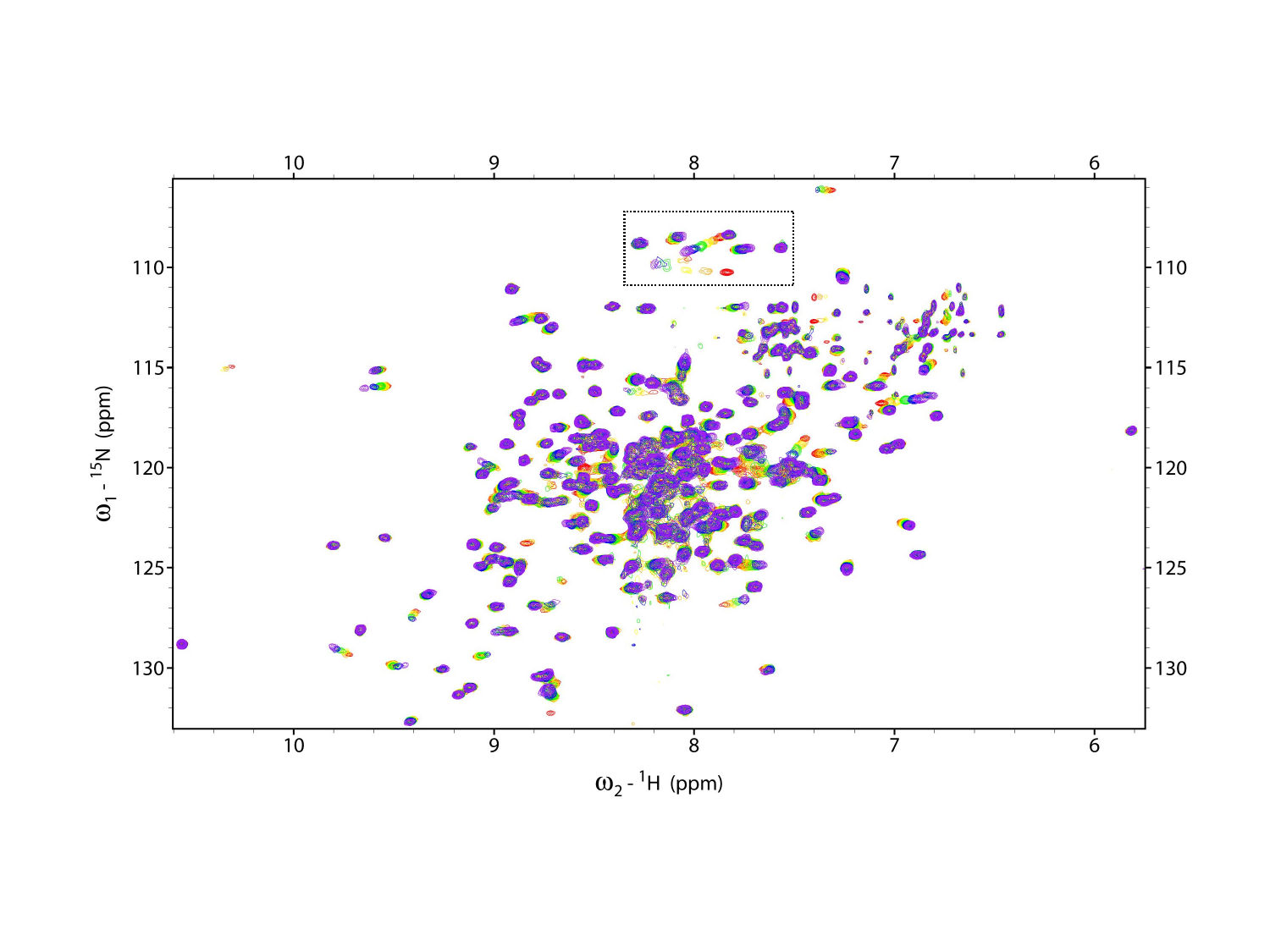

Supplement: Additional file 1 — Fig. S1. Overlay of the full 1H-15N HSQC spectra of PfPMT in the absence (red) or presence of 0.06 (orange), 0.12 (yellow), 0.25 (green), 0.5 (blue) and 1 mM (purple) of AQ. [file 1471-2091-11-4-S1.ppt]
