# Supplementary material for: Identification of inhibitors of Plasmodium falciparum phosphoethanolamine methyltransferase using an enzyme-coupled transmethylation assay
Source: BMC Biochem. 2010 Jan 19;11:4. doi: 10.1186/1471-2091-11-4 (PMC2824672; doi:10.1186/1471-2091-11-4)
Supplement: Additional file 2 — Fig. S2. Sequence alignment of HNMT and PfPMT. Residues that are identical, conserved, and semi-conserved are indicated by asterisk, colon, and period, respectively. The AQ-interacting residues are colored as in Fig. 8B. Phe19 and Tyr198 of HNMT and their corresponding residues in PfPMT are shown in italics and bold. [file 1471-2091-11-4-S2.ppt]

## Slide 1
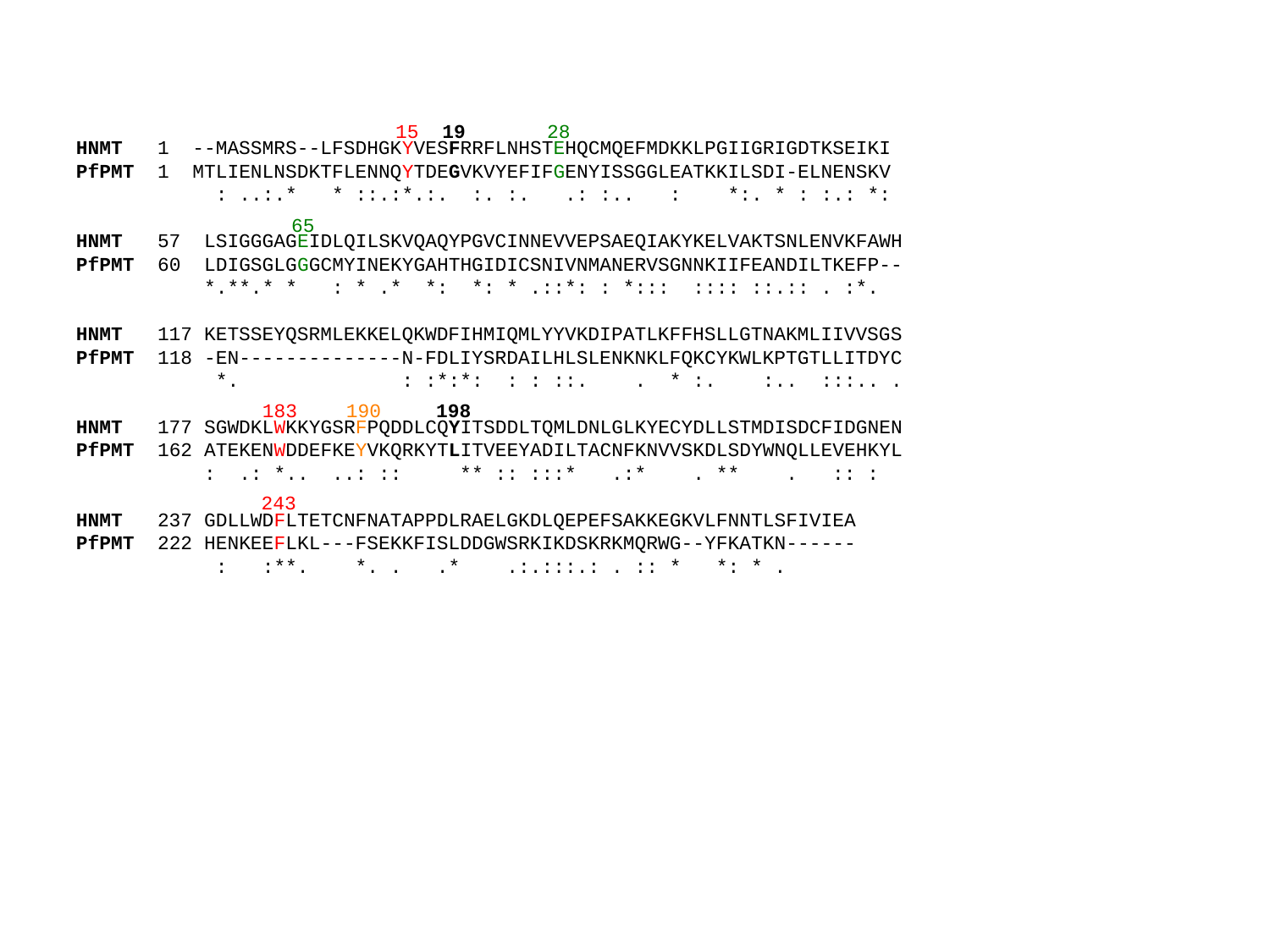

HNMT 1 --MASSMRS--LFSDHGKYVESFRRFLNHSTEHQCMQEFMDKKLPGIIGRIGDTKSEIKI
PfPMT 1 MTLIENLNSDKTFLENNQYTDEGVKVYEFIFGENYISSGGLEATKKILSDI-ELNENSKV
 : ..:.* * ::.:*.:. :. :. .: :.. : *:. * : :.: *:
HNMT 57 LSIGGGAGEIDLQILSKVQAQYPGVCINNEVVEPSAEQIAKYKELVAKTSNLENVKFAWH
PfPMT 60 LDIGSGLGGGCMYINEKYGAHTHGIDICSNIVNMANERVSGNNKIIFEANDILTKEFP--
 *.**.* * : * .* *: *: * .::*: : *::: :::: ::.:: . :*.
HNMT 117 KETSSEYQSRMLEKKELQKWDFIHMIQMLYYVKDIPATLKFFHSLLGTNAKMLIIVVSGS
PfPMT 118 -EN--------------N-FDLIYSRDAILHLSLENKNKLFQKCYKWLKPTGTLLITDYC
 *. : :*:*: : : ::. . * :. :.. :::.. .
HNMT 177 SGWDKLWKKYGSRFPQDDLCQYITSDDLTQMLDNLGLKYECYDLLSTMDISDCFIDGNEN
PfPMT 162 ATEKENWDDEFKEYVKQRKYTLITVEEYADILTACNFKNVVSKDLSDYWNQLLEVEHKYL
 : .: *.. ..: :: ** :: :::* .:* . ** . :: :
HNMT 237 GDLLWDFLTETCNFNATAPPDLRAELGKDLQEPEFSAKKEGKVLFNNTLSFIVIEA
PfPMT 222 HENKEEFLKL---FSEKKFISLDDGWSRKIKDSKRKMQRWG--YFKATKN------
 : :**. *. . .* .:.:::.: . :: * *: * .
15
19
28
65
183
190
198
243
